# Electrocapillary Actuation of Liquid Metal in Microchannels

**DOI:** 10.3390/mi13040572

**Published:** 2022-04-03

**Authors:** Saige J. Dacuycuy, Wayne A. Shiroma, Aaron T. Ohta

**Affiliations:** Department of Electrical and Computer Engineering, University of Hawai’i at Mānoa, Honolulu, HI 96822, USA; wayne.shiroma@hawaii.edu

**Keywords:** electrocapillary actuation, liquid metal, microchannels

## Abstract

Controllable deformation of liquid metal by electrocapillary actuation (ECA) is empirically characterized in fluidic channels at the sub-millimeter-length scale. In 100-µm-deep channels of varying widths, the Galinstan liquid metal could move at velocities of more than 40 mm/s. The liquid metal could extend more than 2.5 mm into the channels at an electrocapillary actuation voltage of 3 V DC. The dynamic behavior of the liquid metal as it moves in the microchannels is described. These results are useful for designing microsystems that use liquid metal as a functional material.

## 1. Introduction

Gallium-based metal alloys such as eutectic gallium-indium and Galinstan are liquid at room temperature and exhibit high electrical conductivity, high thermal conductivity, and low toxicity [1,2,3]. These properties make it possible to integrate liquid metal into antennas [4,5,6], sensors [7,8], thermal management devices [9,10,11], actuation systems [12,13], electronics [14,15], thermoelectrics [16], plasmonics [17,18], and catalysis [19].

In applications such as [5,6] and [9,10,11,12,13], the position or the physical dimensions of the liquid metal is altered via electrical actuation techniques that manipulate the liquid metal’s surface tension. Compared to mechanically pressure-driven methods, electrical actuation is a relatively low power (typically milliwatts or less) [20] technique.

Liquid-metal motion in confined channels using electrical actuation was investigated in [20] and [21]. In [20], a quasi-planar reservoir was used to increase the liquid metal’s sensitivity to an electrical actuation technique called electrocapillary actuation (ECA). In [21], finite volumes of liquid metal entered and exited a confined channel while under the influence of a DC electric field. Although both works demonstrated electrically-driven liquid metals in a confined environment, the device channel widths were 1 mm or larger. However, liquid metal could also be used in smaller channels for potential applications such as millimeter-wave wireless systems [22] or three-dimensional heterogeneous integration [23]. Thus, it is important to observe the dynamics of liquid metal in sub-millimeter-wide channels.

This work investigates ECA-activated liquid metal in channels with sub-millimeter widths. The results presented here will aid in designing devices that require liquid metal with characteristic dimensions in the microscale.

## 2. Materials and Methods

**Figure 1:**
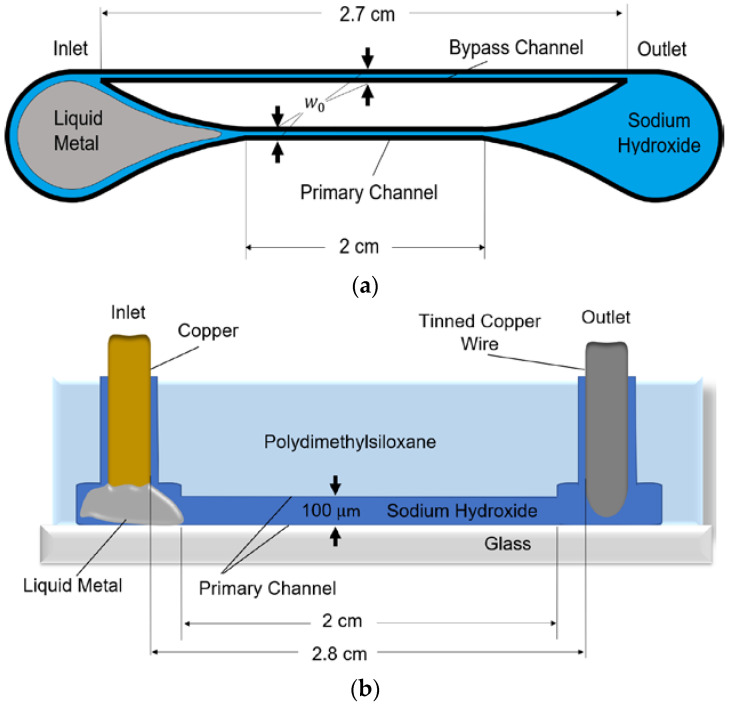
Layout of the microfluidic device for the electrocapillary actuation of liquid metal: (**a**) top view and (**b**) cross-sectional view.

The microchannels used to characterize the liquid metal were fabricated in a layer of polydimethylsiloxane (PDMS) that was bonded to glass (Figure 1). The structure has inlet and outlet reservoirs at the ends of the device. Connecting these reservoirs are two channels of the same width $w_{0}$: a 2-cm-long primary channel that facilitates liquid metal flow and a 2.7-cm-long bypass channel to divert electrolytic fluid displaced by the liquid metal. The bypass channel is longer than the primary channel to increase its fluidic resistance and make the liquid metal flow preferentially into the primary channel. Both channels have a height of 100 μm.

The channels are filled with a 1 M sodium hydroxide (NaOH) solution, and the inlet reservoir contains Galinstan liquid metal. Gallium-based liquid metals form self-passivating oxide skins [24], which wet to many surfaces. The NaOH solution reduces the oxide layer around the liquid metal, which allows the liquid metal to behave like a liquid and respond to the ECA actuation signal.

Since liquid metal readily wets to copper, a copper rod was fixed in the inlet to serve as one electrode. As a result of liquid metal wetting to copper, the liquid metal is bound to the channel inlet. A 22-AWG tinned copper jumper wire was fixed in the outlet to serve as the second electrode; however, the outlet electrode could be made of any conductive material. The distance between the electrodes is 2.8 cm. An Arduino Mega ADK was used to control the ECA electrode voltage.

## 3. Concept

**Figure 2:**
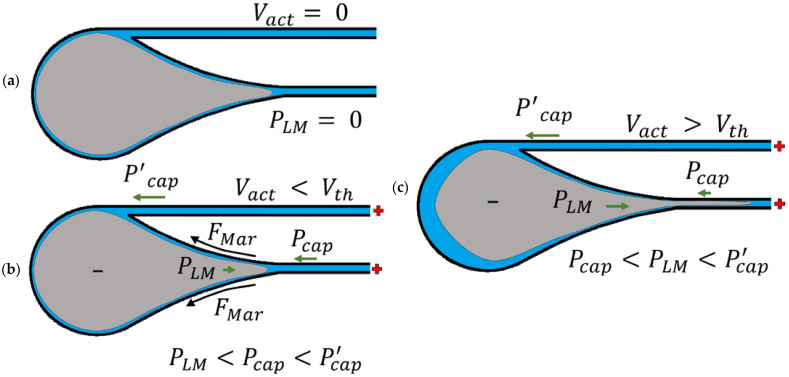
Electrocapillary actuation of liquid metal. (**a**) Liquid metal resting in an oval-shaped reservoir. (**b**) Applying a positive DC voltage ($V_{act}$) creates surface-tension gradients and Marangoni forces ($F_{Mar}$) at and near the surface of the liquid metal. The liquid metal now exerts a positive pressure ($P_{LM}$) on the channel entrances and opposes the channels’ capillary pressure ($P_{cap}$ and $P_{cap}^{\prime }$). (**c**) Once the actuation voltage is above the voltage threshold $V_{th}$, the pressure exerted by the liquid metal exceeds the capillary pressure near the primary channel entrance. The bypass channel has a larger fluidic resistance, so the liquid metal preferentially flows into the primary channel.

The concept is illustrated in Figure 2. If an actuation voltage $V_{act}$ is not applied to the electrodes, the liquid metal is at rest in the inlet reservoir since it does not exert any pressure into the channels. Applying a positive voltage to the NaOH solution relative to the liquid metal creates surface-tension gradients and generates Marangoni forces $F_{Mar}$, allowing the liquid metal to deform and enter the channels. To induce flow into the channels, the pressure exerted by the liquid metal $P_{LM}$ must exceed the channels’ capillary pressure. This condition is met when $V_{act}$ is above some critical voltage threshold $V_{th}$. Since the bypass channel is longer than the primary channel, the capillary resistance of the bypass channel, $P_{cap}^{\prime }$, is larger than the resistance of the primary channel, $P_{cap}$. The liquid metal will move down the path of least resistance, which is the primary channel, when $P_{LM}$ is greater than $P_{cap}$.

**Figure 3:**
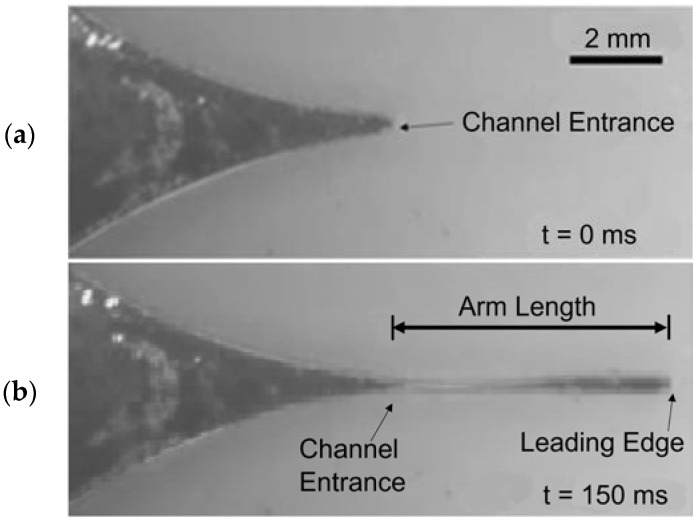
Liquid metal extending into the primary channel through ECA. (**a**) When there is no applied voltage, the liquid metal is resting at the channel entrance. (**b**) The liquid metal flowing in the primary channel. The displacement between the liquid-metal arm’s leading edge and the channel entrance is measured to obtain the liquid-metal arm’s length.

Figure 3 illustrates the ECA mechanism. As the liquid metal enters and then travels down the primary channel, the liquid metal elongates within the channel. The arm length is defined as the displacement from the primary channel’s entrance to the leading edge of the liquid metal.

**Figure 4:**
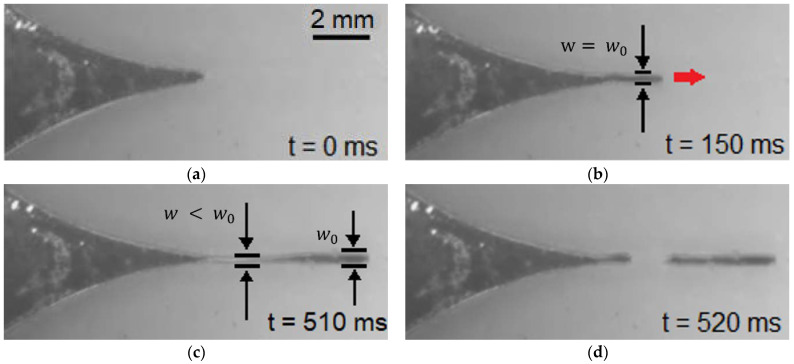
Demonstration of ECA for the liquid metal in a 200-μm-wide channel. (**a**) In the absence of an applied voltage, the liquid metal is initially at rest. (**b**) The liquid metal elongates and flows down the primary channel; this image is taken 150 ms after applying a positive 3 V DC bias. The width of the liquid-metal arm is approximately the same as the channel width. (**c**) At t = 510 ms, the width of the liquid-metal arm gets thinner near the channel entrance and is narrower than the width of the arm at the leading edge, which remains approximately the same as the channel width. (**d**) As the liquid metal moves down the primary channel, the liquid-metal arm breaks into discrete volumes, near the point at which the liquid-metal arm is thinnest.

Figure 4 (Video S1) shows a time sequence for the liquid metal motion in a 200-μm-wide channel. In the following, we define the channel width as $w_{0}$ and the minimum width of the liquid-metal arm as w. Figure 4a shows the liquid metal at rest when no voltage is applied. Figure 4b shows a representative snapshot when the liquid metal has just entered the primary channel, where $w=w_{0}$. Figure 4c shows that as the liquid metal continues to elongate in the primary channel, w decreases such that $w<w_{0}$ at some point (usually near the primary channel entrance) until the liquid-metal arm breaks, as shown in Figure 4d. The maximum arm length is defined as the longest arm length possible before the liquid-metal arm breaks.

## 4. Results and Discussion

### 4.1. Elongation of Liquid Metal

**Figure 5:**
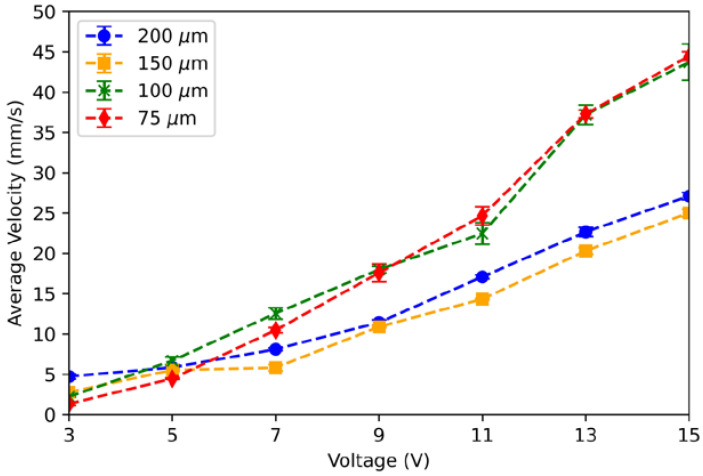
Average ECA velocity as a function of voltage. The narrower channels exhibit higher average velocities than the wider channels as the actuation voltage increases.

The average velocity of the liquid-metal arm as a function of actuation voltage was measured for four channel widths (Figure 5). The average velocity is found by measuring the displacement between the liquid metal at rest to the furthest point before it breaks, then dividing by the time it takes the liquid metal to travel that displacement. A data point in Figure 5 represents the mean of the average velocity for six trials.

For all channels, the average velocity increases as the actuation voltage increases. The liquid metal in the narrower (75 μm and 100 μm) channels exhibit higher actuation velocities compared to liquid metal in the wider (150 μm and 200 μm) channels. For an actuation voltage of 15 V DC, liquid metal in the narrower channels has velocities approximately 1.7 times faster than in the wider channels. Intuitively, the average velocity should saturate as the actuation voltage increases, but the relationship is roughly linear for the voltages used in this experiment.

**Figure 6:**
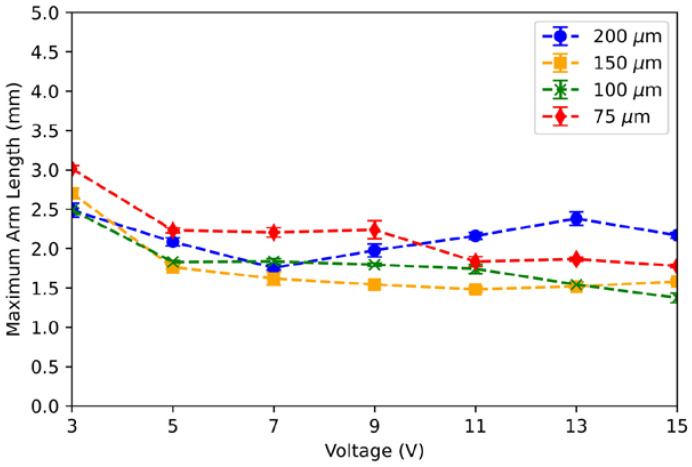
Maximum arm length as a function of voltage. The maximum arm length decreases slightly as the actuation voltage increases.

Figure 6 shows the maximum arm length as a function of voltage. Each data point represents the mean of the maximum arm lengths for six trials. The general trend shows that the maximum arm length decreases as the voltage increases. The likely reason for shorter maximum arm lengths at higher voltages is due to the instability of liquid metal during rapid deformation. When the actuation voltage is low and the liquid metal moves more slowly, the system is more mechanically stable, allowing the liquid metal to stretch to longer lengths. However, as shown in Figure 5, higher voltage causes the liquid metal to move more quickly, decreasing the system’s stability. This likely explains why the liquid metal breaks at shorter arm lengths as the actuation voltage is increased.

### 4.2. Occupation Ratio

**Figure 7:**
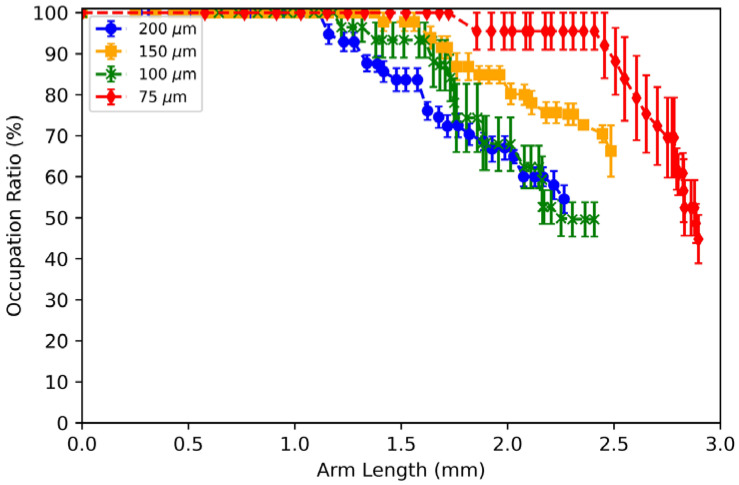
The occupation ratio, or the ratio between the liquid-metal arm’s thinnest width to its respective channel width ($w/w_{0}\times 100\%$), as a function of the arm length. For all channel widths, the liquid-metal arm retains an occupation ratio up to 100% at an arm length of 1 mm and can extend beyond an arm length of 2 mm before breaking. The actuation voltage is set at 3 V DC.

To provide more insight into the liquid-metal breakup dynamics shown in Figure 4, we define the occupation ratio, w/$w_{0}\times 100\%$. Figure 7 plots the measured occupation ratio as a function of the arm length. For this measurement, the actuation voltage was set at 3 V DC so that the liquid-metal arm could extend to its greatest length. Each data point represents the average of six trials, but the arm length was only averaged up to the shortest maximum arm length among the six trials. Thus, the end of the curves in Figure 7 may not have the same arm length value shown in Figure 6.

For all channel widths, the liquid-metal arm retains an occupation ratio of 100% up to an arm length of 1 mm (Figure 7). For all channel widths tested, the liquid metal’s occupation ratio decreases between an arm length of 1 mm to 1.5 mm. The liquid metal’s occupation ratio then decreases by 40% per mm, regardless of the channel width. This shows that the liquid-metal arm for all channel widths can undergo sizable deformation before reaching its breaking point.

If the liquid-metal arm breaks apart during ECA, the voltage polarity can be switched to return the broken segment of liquid metal into the inlet reservoir. The ability to return the liquid metal back to its original state suggests that the device can be used multiple times.

**Figure 8:**
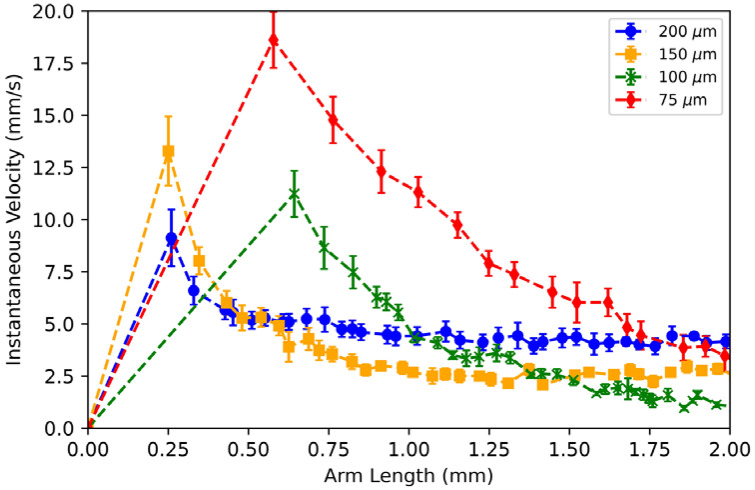
Measured ECA actuation velocities for an actuation voltage of 3 V DC. For all channel widths, as the liquid-metal arm increases, the instantaneous velocity reaches a maximum value and gradually decreases.

The instantaneous velocity at the liquid-metal arm’s leading edge was also measured when a 3-V DC ECA voltage was applied (Figure 8). The arm length was observed up to 2 mm since the liquid metal in all channels remains unbroken at this arm length. Like Figure 7, a data point in Figure 8 represents the average of six trials, except the arm length was averaged only up to the shortest maximum arm length among the six trials.

For all channel widths, as the liquid-metal arm increases, the instantaneous velocity reaches a maximum value and then gradually decreases, showing that the instantaneous velocity is not constant as the liquid metal moves down the channel.

## 5. Conclusions

In summary, this paper characterizes the deformation of liquid metal in microchannels due to ECA. The dynamics and the physical limitations of actuated liquid metal were observed for a voltage range of 3 V DC to 15 V DC. At an actuation voltage of 3 V DC, the liquid-metal arm could extend more than 2.5 mm into the channel. Although the results presented here were specific to a 2-cm long channel, the results could be generalized for channels of other lengths by converting the ordinate axes of Figure 5 and Figure 6 to an electric field, which is the actuation voltage divided by the distance between the electrodes (2.8 cm). The results presented from this research will aid in the design of microdevices that use electrically actuated liquid metal, such as biomedical devices and sub-millimeter systems that require reconfigurable and tunable metal elements. For example, the deformation of liquid metal at these small volumes could be used in millimeter-wave systems. Adjusting the length of a liquid-metal arm could be used to create a tunable load-matching transmission line stub.

## Data Availability

The data presented in this study are available upon request.

## Supplementary Materials

The following supporting information can be downloaded at: https://www.mdpi.com/article/10.3390/mi13040572/s1. Video S1: Demonstration of ECA for the liquid metal in a 200-μm-wide channel. The blue and red markers represent the minimum width of the liquid-metal arm and the channel width, respectively.
